# Two-Year Outcome of Aflibercept in Patients with Pigment Epithelial Detachment due to Neovascular Age-Related Macular Degeneration (nAMD) Refractory to Ranibizumab

**DOI:** 10.1155/2017/8984313

**Published:** 2017-09-13

**Authors:** Thi Ha Chau Tran, Stéphane Dumas, Florence Coscas

**Affiliations:** ^1^Ophthalmology Department, Lille Catholic Hospitals, Lille Catholic University, Lille, France; ^2^Clinique de la Louvière, Lille, France; ^3^Centre Ophtalmologique de l'Odéon, Université Paris-Est Créteil (UPEC), Paris, France

## Abstract

**Purpose:**

To evaluate the response of intravitreal aflibercept injection (IAI) in eyes with detachment of retinal pigment epithelium (DEP) secondary to nAMD refractory to monthly ranibizumab.

**Patients and Methods:**

This is a retrospective, multicenter study. All patients received 3 IAI then treated as needed every 4 weeks for 12 months. During the second year, the eyes were treated with a treat- and-extend regimen.

**Results:**

Forty-four eyes were included. Best-corrected visual acuity improved significantly after the loading phase (3.1 ± 6.4 letters) and at 6 months (2.8 ± 6.4 letters), but change was not significant at 1 year and 2 years. The height of the DEP was significantly decreased at 3 months and 6 months, but the difference did not reach statistical difference at 1 and 2 years. Rate of eyes with complete resolution of exudation was 59% after the loading phase and 34.3% at 2 years. Mean interval of anti-VEGF injection was extended from 31 ± 2.6 days to 61 ± 5 days after conversion.

**Conclusions:**

Aflibercept intravitreal injection in patients with fibrovascular DEP due to nAMD who respond poorly to monthly ranibizumab led to short-term functional and anatomical improvement. Reduction of intravitreal injection frequency was obtained until 2 years of follow-up.

## 1. Introduction

Neovascular age-related macular degeneration is characterized by choroidal neovascularization (CNV), which lead to the accumulation of intraretinal fluid (IRF), subretinal fluid (SRF), and pigment epithelium detachment (PED). The prognosis of nAMD has improved considerably with intravitreal (IVT) injections of anti-VEGF [1]. Ranibizumab, a fragment binding to the monoclonal antigen VEGF-A without an Fc fragment, neutralizes all the active isoforms of VEGF-A [1]. The efficacy and safety of ranibizumab has been demonstrated in the MARINA, ANCHOR, and CATT studies, and this product was approved for the treatment of nAMD in Europe in January 2007 [1–4]. Aflibercept, approved in Europe in November 2012, was available and reimbursed in exudative AMD in France since November 2013. Aflibercept is a fusion protein that combines VEGF receptor 1 and 2 fragments (VEGFR1, VEGFR2) with an Fc fragment. It binds to VEGF-A, VEGF-B, and placental growth factor (PIGF). After the induction period, bimonthly intravitreal aflibercept injection (IAI) has been shown to be safe and effective as ranibizumab monthly injection in the treatment of nAMD in phase III of VIEW 1 and VIEW 2 studies [5].

Pigment epithelial detachments (PEDs) have been identified in up to 66.5% eyes in nAMD and are generally associated with poor visual prognosis, with loss of more than 3 lines in approximately 50% of patients within 1 year [6]. Clinical trials in nAMD have either excluded eyes with PED or have not performed subanalysis of PED response to treatment [2–4, 7]. Although anti-VEGF is the standard care of nAMD [2–4, 8, 9], some cases are refractory with persistent fluid, and others develop a tolerance or tachyphylaxis defined by a decrease in anatomical response over time while they respond initially to treatment [10].

Pharmacological studies have shown that aflibercept differs from bevacizumab and ranibizumab by its higher affinity and additionally inhibits placental growth factor (PIGF) [11]. These differences led to several studies on the advantages of switching from ranibizumab and/or bevacizumab to aflibercept in refractory nAMD cases and its potential for the treatment of PED-related nAMD [12–17]. With the exception of some studies [18–21], most studies showed anatomical [15, 17, 22–28] but usually no functional benefit.

The purpose of the study was to evaluate the intravitreal aflibercept for the treatment of type 1 choroidal neovascularization-related PEDs with persistent exudation despite monthly ranibizumab during the 12 months preceding conversion with at least 2 years of follow-up.

## 2. Patients and Method

This is a retrospective, multicenter, nonrandomized study which recruited patients with PEDs due to type 1 choroidal neovascularization (CNV) who had been treated with monthly ranibizumab for at least 12 months in 3 centers (Centre Ophtalmologique de l'Odéon, Paris, France; Clinique de la Louvière, Lille, France; and the Ophthalmology Department, Catholic University of Lille, France). Informed consent was obtained from all participants. The study was conducted in accordance with the Declaration of Helsinki, and all applicable institutional and governmental regulations concerning the ethical use of human volunteers were followed during this research. This study named “OPENN” (a noninterventional Observational retrosPective study to access the Efficacy of intravitreal injections of aflibercept in NonNaïve patients with fibrovascular retinal pigment epithelium detachment secondary to wet AMD) had obtained governmental regulations with the reference MMS/SBA/AR149432.

Inclusion criteria were as follows: (1) eyes with PEDs related to type 1 CNV refractory to monthly ranibizumab (defined as the presence of intraretinal fluid (IRF) or subretinal fluid (SRF) at each visit 1 month after injection) during the 12 months leading to the switch from ranibizumab to aflibercept between November 2013 to April 2014, (2) baseline best-corrected visual acuity (BCVA) score between 20/25 and 20/250, and (3) last ranibizumab injection less than 3 months prior to the initiation of aflibercept injections (2 mg/0.05 ml). The PEDs secondary to exudative AMD were defined by the presence of subfoveolar occult CNV and vascularized DEP on fluorescein angiography (pinpoint leakage), hypofluorescence of DEP and choroidal neovascular visualization on indocyanine green angiography, and hyporeflective DEP on optical coherence tomography (SD-OCT) [6].

Exclusion criteria were the history of intraocular surgery, intraocular inflammation during the 3 months prior to initiation of aflibercept therapy, history of triamcinolone acetonide IVT therapy, macular laser photocoagulation or photodynamic therapy, PED due to a cause other than AMD, and occult neovascular lesions without DEP or any other active retinal disease.

Measurement of Early Treatment Diabetic Retinopathy Study (ETDRS), best-corrected visual acuity (BCVA), intraocular pressure assessment, spectral-domain optical coherence tomography (SD-OCT), fluorescein angiography, and indocyanine green angiography using a confocal laser scanning ophthalmoscope (HRA2; Heidelberg Engineering GmbH, Heidelberg, Germany) were performed at baseline. Visual acuity, adverse event monitoring, and SD-OCT were recorded at each visit using 49 line cube examination of Spectralis. The SD-OCT-derived images had been obtained by using an eye-tracking system. Inverted images had also been routinely obtained by enhanced depth imaging technique (EDI) [29]. Central retinal thickness (CRT) and macular (MT) volume were computed automatically by the software (Heidelberg Eye Explorer, Heidelberg, Germany). Maximum PED height was measured on SD-OCT imaging using the built-in caliper tool. Maximum height was defined as the distance from underneath the hyperreflective pigment epithelium band perpendicular to the Bruch's membrane on SD-OCT on a 1 : 1 scale, including extrafoveal location. Subfoveal choroidal thickness (CT) was defined as the vertical distance between the Bruch's membrane and the chorioscleral scale using EDI-OCT through the center of the fovea. PED height and subfoveal choroidal thickness were manually measured. Analyses of OCT scans and variables measurements were conducted by an ophthalmologist (AB) masked to the patient's characteristics. The presence of IRF, SRF, hyperreflective subretinal exudation (HSE), and disruption of inner segment/outer segment (IS/OS) was defined as previously [30, 31].

All patients received 3 monthly intravitreal of aflibercept at baseline (2 mg/0.05 ml) and then were scheduled for monthly monitoring visits including an ETDRS score measurement and SD-OCT [32]. After the loading phase, patients were treated with pro re nata (PRN) regimen during the first year and “treat and extend” regimen during the second year. Additional imaging was planned upon the physician's discretion. Reinjection criteria were the presence of IRF or SRF on SD-OCT but not fluid immediately underneath the PED. During the second year, treatment interval was extended in case of absence of IRF or SRF.

Data such as demographic characteristics, history of disease, history of ranibizumab treatment, and follow-up duration before and after the switch were collected from medical records and entered into an electronic file. ETDRS score, central macular thickness (CMT), macular volume, subfoveal choroidal thickness, the maximum height of the PED (from the Bruch's membrane to the posterior surface of the pigmentary epithelium), the presence of intraretinal fluid (IRF), subretinal fluid (SRF), subretinal hyperreflective exudations (SHE), and disruption of the IS/OS (Inner/Outer segment) zone were collected. Data were collected monthly from baseline to 6 months, at 12 months and at 24 months.

The statistical analysis was performed as paired comparisons between different time points using SPSS for Windows (version 17.0/SPSS Inc., Chicago, IL). The paired *t*-test and Wilcoxon were used for comparison between paired continuous variables, and *t*-test and Mann–Whitney *U* test were used for comparison between subgroups. Statistical significance was set as *p* < 0.05.

## 3. Results

A total of 44 eyes of 44 patients with PEDs due to nAMD previously treated with at least 12 intravitreal ranibizumab injections during the last 12 months were switched to aflibercept therapy. Visual acuity from the 12-month period before enrollment was available for all patients. Mean BCVA was 67 ± 12.2 12 months before switching therapy and 64.4 ± 13 at baseline. There was a trend for worsening of BCVA (−2.6 ± 9.4, *p* = 0.07) during the 12-month preinclusion period.

Baseline (time of switch from ranibizumab to aflibercept) characteristics of patient cohort are summarized in Table 1. Thirty patients were female (68.2%), and the mean age was 78.5 ± 9.5 years. Duration of PED history was 43 ± 3.6 months at time of switch, and 27.8 ranibizumab injections have been given during this period. During the year preceding the medication switch, mean of 12 ± 1 injections had been given. Distribution of fluid was as followed: IRF in 22 eyes (50%), SRF in 15 eyes (11.4%), and both intra- and subretinal fluid in 15 eyes (34%). Eighteen eyes (40.9%) displayed hyperreflective subretinal exudation, and 19 eyes (43.2%) had IS/OS segment zone disruption.

### 3.1. Adverse Events

Adverse events were reported in 2 patients. A transient ischemic attack occurred at month 5 in one patient. Multiple myelomas were discovered in another patient at month 6. Three patients were lost to follow-up from month 6 to month 12. Two eyes had evidence of macular atrophy at 1 year. Three patients were dead and 3 others were lost to follow-up from month 12 to month 24. Three eyes developed progression of cataract during the second year. At the end of the study, 38 patients reached the end point of 2 years.

### 3.2. Functional Response to Aflibercept

Change in visual acuity and OCT parameters were summarized in Table 2.

**Figure 1:**
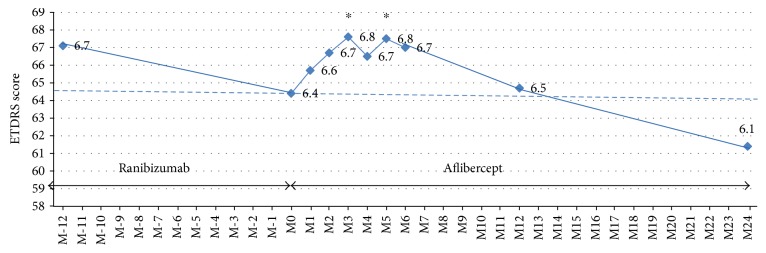
Visual acuity change over visits. ETDRS: Early Treatment Diabetic Retinopathy Study scale for visual acuity; M-12: 12 months before switching from ranibizumab to aflibercept; M0: time of switch from ranibizumab to aflibercept; M3: 3 months after switch; M6: 6 months after switch; and M12: 12 months after switch. ^∗^*p* < 0.05.

A statistically significant improvement in BCVA was reported at month 3 (+3.2 letters) (from 64.4 ± 13 to 67.6 ± 12, *p* = 0.002) and at month 6 (+2.84 letters) (67.3 ± 11.2, *p* = 0.005). At month 12 and month 24, visual change was not significant compared to baseline (64.8 ± 13.3, *p* = 0.6 and 61.4 ± 13.9, *p* = 0.18). After 2 years of aflibercept treatment, 2 eyes (4.8%) displayed ≥15 letters gain, no patient had 10–14 letters gain, 3 patients (13.7%) had 5–9 letters gain, 3 patients (13.7%) had 0–4 letters gain, and 4 (24.3%) patients had experienced ≥15 letters loss. Visual acuity evolution was illustrated in Figure 1.

### 3.3. Anatomical Response to Aflibercept

#### 3.3.1. Central Retinal Thickness (CRT) and Macular Volume (MV)

CRT was 313 ± 85 *μ*m at baseline decreased to 301 ± 77.5 at M3 after the loading phase (−12 ± 67 *μ*m, NS). Change of CRT did not reach the significant difference at any time point of the study (M6: 312 ± 91 *μ*m, NS; M12: 312 ± 84 *μ*m, NS; M24: 312 ± 12.3, NS). MV did not change from baseline to any time point during the 2 years of follow-up (7.7 ± 1.4 at baseline; 7.75 ± 1.1 at month 3; 7.8 ± 1.11 at month 6; 7.8 ± 1.2 at month 12; and 7.6 ± 1.44, NS).

#### 3.3.2. Distribution of Fluid and Qualitative SD-OCT Analysis

Distribution of fluid on SD-OCT was summarized in Table 3. IRF and/or SRF were present in all eyes at the beginning of the study. At month 3, 26/44 eyes (59%) displayed complete resolution on SD-OCT and 12/35 eyes (34.3%) had complete resolution of exudation at 2 years end- point. SHE was present in 18 eyes (43.2%) at baseline, in 2 eyes (4.5%) at month 3 and in 5 eyes (13.1%) at 2 years. IS/OS disruption was observed in 19/44 eyes (43.2%) at baseline, in 17/44 eyes (38.6%) at month 3 and month 6, then the rate increased to 29/44 (65.9%) eyes at one year and in 30/38 (78.9%) at 2 years. Visual change was −1.4 letters in 8 eyes without IS/OS disruption and −2.2 letters in eyes with IS/OS disruption.

#### 3.3.3. Pigment Epithelium Detachment Response to Aflibercept

**Figure 2:**
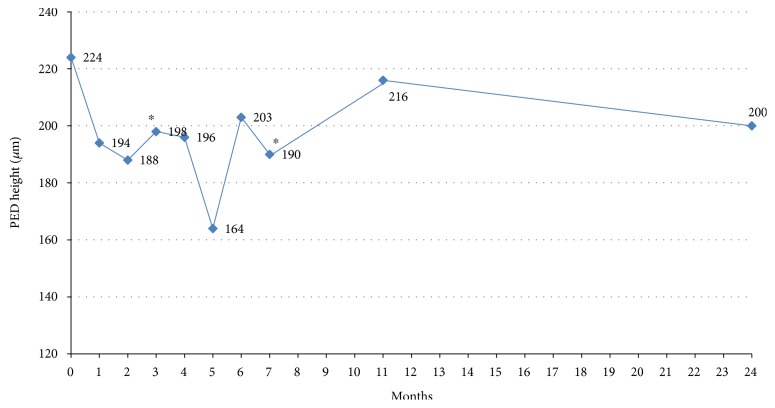
PED height change over visits from month 0 to month 24. M0: time of switch from ranibizumab to aflibercept. Significant decrease of mean PED height was found at 3 months and 6 months after switch (^∗^*p* < 0.05). Change was not significant at 12 months and 24 months.

A decrease in mean PED height compared to baseline was observed at month 3 (from a preaflibercept mean of 224 ± 18.5 *μ*m to 198 ± 19.5 *μ*m −26.6 *μ*m, *p* = 0.025, after the loading phase) and to 190.4 ± 17.4 *μ*m at month 6 (−28.1 *μ*m). However, change in PED height was not significant at 1 year (216 ± 17.9 *μ*m) and 2 years (200 ± 19.5 *μ*m). A PED height reduction of at least 20% was achieved in 13/44 (29.5%) eyes after the loading phase, in 9/36 (25%) eyes at 1 year and in 9/37 (24.3%) eyes at 2 years. In three eyes, the PED was flattened at the last follow-up; however, visual acuity decreased in these eyes (−8 to −25 letters) because of macular atrophy. PED height evolution was illustrated in Figure 2.

#### 3.3.4. Subfoveal Choroidal Thickness

Subfoveal choroidal thickness was available at baseline, 12 months, and 24 months. It decreased from 179 ± 68 *μ*m at baseline to 158 ± 67 *μ*m at month 12 with a mean change of −25 *μ*m, then increased to 174 ± 56 *μ*m (NS from baseline, *p* = 0.5).

### 3.4. Frequency of Anti-VEGF Intravitreal Injection

Mean time between the last injection of ranibizumab and the first injection of aflibercept was 2.0 ± 1.2 months. Mean interval of ranibizumab injections during the year before medication switch was 31 ± 2.6 days. Mean number of intravitreal aflibercept injection was 7.6 ± 0.6 during the first year (including the 3 monthly injections at the loading phase) and 6.9 ± 0.6 during the second year. Mean interval of intravitreal aflibercept injection, from the end of the loading phase and 1 year time point, was 60.9 ± 5 days (ranging from 28–135) and 61.5 ± 5 (ranging from 31.8 ± 155) days during the second year, which was longer than the mean interval injection before medication switch (*p* < 0.0001). Two eyes needed monthly aflibercept injection, and stepwise from Q8W to Q4W occurred between 9 months and 1 year after medication change. The average number of injections was reduced approximately by 0.6 compared with the 12 months before the switch.

## 4. Discussion

This retrospective, observational study was designed to evaluate the efficacy and safety of switch from ranibizumab to aflibercept in patients with PEDs related to nAMD with persistent exudation despite monthly ranibizumab injection during the 12-month period preceding inclusion. The results showed that medication switch, even conducted after 43 month duration of PEDs, led to a short-term improvement in both functional and anatomical response: significant moderate visual gain at 3 months (+3 letters) and at 6 months (+2.6 letters), decrease of maximum PED height at 3 months (−26.6 *μ*m) and at 6 months (−28.1 *μ*m), and decreased rate of eyes with IRF, SRF, and SHE until 6 months after switch. The benefit was then less significant with time course at 1 and 2 years. At the end of 2 year follow-up, visual acuity remained stable compared to baseline, and 34.4% eyes achieved a dry macula. Choroidal thickness decreased at one year, but this effect disappeared at 2 years. The frequency of anti-VEGF injections dropped from 12 ± 1 the year preceding medication switch to 7.6 ± 0.6 during the first year and 6.9 ± 0.6 during the second year. Treatment interval was lengthened from 31 ± 2.6 days to 61 ± 5 days after the loading phase then remained unchanged with time course. Overall, these patients could have anatomic improvement, and the injection intervals could be extended.

Various studies have examined the efficacy of aflibercept in patients with PED refractory of ranibizumab and/or bevacizumab treatment [17, 18, 20, 23, 33–35]. However, comparisons are difficult due to many differences: duration of anti-VEGF varied from 3 months to 12 months before switch, inclusion of vascularized PEDs [17, 20, 33] and/or serous PEDs; [15] visual acuity was expressed in ETDRS and logMAR; and PED improvement expressed in maximum height, volume [21], and diameter [20]. Mean follow-up varied from 3 months to 1 year. In these reports, switching from ranibizumab to aflibercept result to unchanged visual acuity in some retrospective studies [15, 18, 26, 28], whereas visual gain had been demonstrated at 6 months or 12 months end point in other prospective studies [17, 19, 35] or retrospective studies with short-term results at 3 months or 6 months [20, 21]. Our study reports results at different time points from baseline to 2 years of aflibercept treatment for PEDs related to nAMD with persistent exudation despite monthly ranibizumab the year prior conversion. We found that functional response under anti-VEGF therapy may change with time course: indeed, in this population with 44 months PEDs history duration, there was a trend toward worse visual acuity from 12 months prior to time of switch (−2.6, *p* = 0.07), and slight visual gain was obtained during 6 months after conversion to aflibercept (+3.2 at month 3 and +2.8 at month 6). Visual acuity decreased after this period and became unchanged at 1 year compared to baseline, and there was a nonsignificant loss of −2.2 letters at 2 years. Visual loss with time course may be explained by retinal structure damage due to chronicity of disease (mean duration of PEDs to switch was 44 months, IS/OS segment disruption found in 78.9% at 2 years in our study) or recurrence of exudation with time with increase rate of eyes with IRF and SRF. This could also indicate a newly developing tachyphylaxis.

Significant improvement in DEP height at month 3 and month 6 in our study is consistent with previously published data showing that treatment with aflibercept led to significant anatomic improvements in patients with persistent exudation under other anti-VEGF [15, 17–20, 23, 28, 33]. Change of PED height was not found at 1 and 2 years, which might be explained by manual measurement of maximum height and absence of inferior limit of PED height in inclusion criteria in our study. Indeed, 1/4 to 1/3 of eyes had reduction of at least 20% over time, which was similar to other reports [15].

The absence of significant changes in the central retinal thickness in our study is surprising. Reduction on CRT thickness varied from −19 *μ*m to −68 *μ*m after a six-month period of aflibercept therapy in published data [17, 18, 20, 23, 35]. These data may reflect variations in patient populations between studies or the fact that our study did not fully meet the power requirements to demonstrate a statistically significant difference. We also found temporary change in the choroidal thickness under aflibercept at one year, in accordance with most of the publications reporting a decrease in the choroidal thickness under aflibercept in naive and switched eyes with nAMD [36, 37].

Aflibercept treatment frequency is a point that needs to be investigated. In this cohort, all patients were treated with >10 injections of ranibizumab in the year prior to conversion, so there was no under treatment before switch. We achieved stabilization of visual acuity with a mean 7.6 injections in the first year (including 3 monthly aflibercept injection of the loading phase) and 6.9 injections during the second year, and mean interval injections was extended from 31 days to 61 days. We did not observe extended interval injection between the first and the second year. Veritti et al. reported an average of only 0.3 ± 0.1 injections per eye per month using pro re nata regimen without loading phase (3.6 ± 1.7 injections for 12 months) after medication switch in a prospective study including 32 eyes with nAMD-related PED, while these patients were administered a total of 4.5 ± 1.2 ranibizumab in the 6 months prior to changing therapy to aflibercept. The frequency was approximately 0.6 injections per eye per month in our study, which is consistent with Singh et al. who found a frequency of 0.7 after medication change [35]. Messenger et al. observed a decreased injection frequency with aflibercept only in patients who received at least 10 injections in the prior 12 months (three fewer injections per year on average), as well as improvement of anatomical outcomes. This suggests that patients with PEDs who required monthly ranibizumab would benefit from transition with extended interval treatment regimen offered by aflibercept [34, 38]. This switch benefit seemed likely because the higher binding affinity of aflibercept [11] and its theoretically longer ligand-binding activity [39]. However, switching from ranibizumab to aflibercept did not reduce the need for retreatment without a selection of refractory cases [16, 34, 40].

This study has limitations of the retrospective nature; the manual measurement of PED height and choroidal thickness and the absence of the control group continuing monthly ranibizumab. It has the advantage of homogenous lesion characteristics (PEDs due to type 1 choroidal neovascularization), homogenous interval between the last injection of `ranibizumab and first injection of aflibercept which may influence the amount of fluid at the time of medication change, and standardized aflibercept regimen.

## 5. Conclusions

In patients with choroidal neovascularization type I associated with a fibrovascular DEP with persistent exudation despite monthly ranibizumab, conversion to aflibercept led to short-term functional and anatomic improvement until 6 months and preserved visual function until 2 years. In this particular form of nAMD, transitioning to aflibercept was associated with a reduced injection frequency, suggesting potential cost saving in this population. Further, prospective study with control groups is needed to determine the benefit of switching from ranibizumab to aflibercept.

## Figures and Tables

**Table 1:** Characteristics of the patient cohort at baseline.

Study eye, *n*	44
Mean age, mean ± SD, range, years	78.5 ± 9.5 (54–98)
Gender distribution, male/female	14/30 (31.8/68.2%)
Mean follow-up before switch ± SD, months	43 ± 3.6
Mean number of ranibizumab injections in the 12 months before enrollment, mean ± SD	12 ± 1
BCVA, ETDRS letters, mean ± SD (range)	64.5 ± 13 (35–80)
Central retinal thickness, *μ*m, mean ± SD, (range)	313 ± 85 (185–508)
PED height, *μ*m, mean ± SD, range	221 ± 120 (38–518)
Intraretinal fluid, *n* (%)	22 (50%)
Subretinal Fluid, *n* (%)	26 (59%)
Intra- and subretinal fluid	15 (34%)
Hyperreflective subretinal exudation	18 (40.9%)
IS/OS segment disruption	19 (43.2%)

BCVA: best-corrected visual acuity; ETDRS: Early Treatment Diabetic Retinopathy Scale; PED: pigment epithelial detachment.

**Table 2:** Comparison of functional and morphologic changes from time of switch to 24 months.

Central macular thickness (*μ*m)	Baseline *N* = 44	Month 3 *N* = 44	*p*	Month 6 *N* = 44	*p*	Month 12 *N* = 44	*p*	Month 24 *N* = 38	*p*
ETDRS	64.4 ± 13.1	67.6 ± 12.3	0.002	67.3 ± 11	0.005	64.8 ± 13	0.6	61.4 ± 13.8	0.2
Central macular thickness (*μ*m)	313 ± 13	312 ± 91	0.2	312 ± 14.2	0.9	313 ± 13.9	0.9	305 ± 76	0.6
Macular volume (mm^3^)	7.70 ± 2.2	7.75 ± 0.16	0.8	7.8 ± 0.16	0.6	7.85 ± 0.16	0.4	7.8 ± 0.65	0.3
PED height (*μ*m)	224.7 ± 18.5	198.5 ± 19.5	0.25	190 ± 17.3	0.5	224 ± 21	0.27	200 ± 19	0.14
Subfoveal choroidal thickness (*μ*m)	179 ± 68					158 ± 67	0.2	174 ± 56	0.5

Data are mean ± SD unless indicated otherwise. *p*: continuous variables compared by independent samples *t*-test from baseline; BCVA: best-corrected visual acuity; ETDRS: Early Treatment Diabetic Retinopathy Scale; PED: pigment epithelial detachment.

**Table 3:** Qualitative analysis of SD-OCT at different time points.

Number of eyes	Month 0	Month 4	Month 6	Month 12	Month 24
	*N* = 44	*N* = 44	*N* = 43	*N* = 38	*N* = 38
SRF	26 (59%)	13 (29.5%)	16 (37.2%)	18 (47.3%)	17 (44.7%)
IRF	22 (50%)	9 (20.4%)	9 (20.9%)	20 (52.6%)	16 (42.1%)
No fluid	0 (0%)	18 (59%)	23 (53.4%)	10 (26.3%)	13 (34.3%)
SHE	18 (40.9%)	2 (4.5%)	1 (2.3%)	5 (13.1%)	5 (13.1%)
IS/OS disruptions	19 (43.2%)	17 (38.6%)	16 (37.2%)	17 (44.7%)	30 (78.9%)

SRF: subretinal fluid; IRF: intraretinal fluid; SHE: subretinal hyperreflective exudation; IS/OS: inner segment/outer segment.
