# Corrigendum to “Two-Year Outcome of Aflibercept in Patients with Pigment Epithelial Detachment due to Neovascular Age-Related Macular Degeneration (nAMD) Refractory to Ranibizumab”

**DOI:** 10.1155/2018/9171269

**Published:** 2018-07-08

**Authors:** Thi Ha Chau Tran, Stéphane Dumas, Florence Coscas

**Affiliations:** ^1^Ophthalmology Department, Lille Catholic Hospitals, Lille Catholic University, Lille, France; ^2^Clinique de la Louvière, Lille, France; ^3^Centre Ophtalmologique de l'Odéon, Université Paris-Est Créteil (UPEC), Paris, France

In the article titled “Two-Year Outcome of Aflibercept in Patients with Pigment Epithelial Detachment due to Neovascular Age-Related Macular Degeneration (nAMD) Refractory to Ranibizumab” [1], there were errors, which should be corrected as follows:

- In Abstract, the term “(DEP)” should be “(PED).”
- In Results, “Change of CRT did not reach the significant difference at any time point of the study (M6: 312 ± 91 *µ*m, NS; M12: 312 ± 84 *µ*m, NS; M24: 312 ± 12.3, NS)” should be corrected to “Change of CRT did not reach the significant difference at any time point of the study (M6: 312 ± 14 *µ*m, NS; M12: 313 ± 13.9 *µ*m, NS; M24: 305 ± 76, NS).”
- In Table 2, there were errors in the values in the third, fourth, and sixth columns. The corrected table and its description are shown below.

## Figures and Tables

**Table 2:** Comparison of functional and morphologic changes from time of switch to 24 months.

Central macular thickness (*µ*m)	Baseline	Month 3	*P*	Month 6	*P*	Month 12	*P*	Month 24	*P*
	*N*=44	*N*=44		*N*=44		*N*=44		*N*=38	
ETDRS	64.4 ± 13.1	67.6 ± 12.3	0.002	67.3 ± 11	0.005	64.8 ± 13	0.6	61.4 ± 13.8	0.2
Central macular thickness (*µ*m)	313 ± 13	301 ± 77	0.2	312 ± 14.2	0.9	313 ± 13.9	0.9	305 ± 76	0.6
Macular volume (mm^3^)	7.70 ± 2.2	7.75 ± 0.16	0.8	7.8 ± 0.16	0.6	7.85 ± 0.16	0.4	7.8 ± 0.65	0.3
PED height (*µ*m)	224.7 ± 18.5	198.5 ± 19.5	0.025	190 ± 17.3	0.05	224 ± 21	0.27	200 ± 19	0.14
Subfoveal choroidal thickness (*µ*m)	179 ± 68					158 ± 67	0.2	174 ± 56	0.5

Data are mean  ±  SD unless indicated otherwise. *P*: continuous variables compared by independent samples *t*-test from baseline; ETDRS: Early Treatment Diabetic Retinopathy Score; PED: pigment epithelial detachment.
